# Radioadaptive Response in Human Lymphocyte Cells

**Published:** 2014

**Authors:** Najmeh Assadi, Ebrahim Zabihi, Meysam Khosravifarsani, Soraya Khafri, Haleh AkhavanNiaki, Mehrangiz Amiri, Ali Shabestani-Monfared

**Affiliations:** 1 *Cellular and Molecular Biology Research Center, Babol University of Medical Sciences, Babol, Iran.*; 2 *Social Medicine and Health Department, Babol University of Medical Sciences, Babol, Iran.*; 3 *Department of Nuclear Medicine, Paramedical Faculty, Babol University of Medical Sciences, Babol, Iran.*

**Keywords:** Adaptive Response (AR), challenge treatment (CR), condition treatment (CT), micronuclei assay

## Abstract

The adaptive response (AR) is a phenomenon by which cells exposure to sublethal doses of DNA-damaging agents (non-mutagenic dose of chemical or radiation), known as conditioning treatment (CT), leads to increased resistance to a subsequent exposure to a higher dose of the same or other agents, known as challenge treatment (CR). The adaptive response (AR) induced by radiation in human lymphocytes has been reported in a range of 1-20cGy pre-exposure. In this study, we investigated the adaptive response using 5cGy conditioning dose of gamma rays followed by 2 Gy challenging dose in peripheral human lymphocyte cells. Blood samples were taken from 30 female volunteers and this experiment was carried out by delivering 5 cGy gamma radiation followed by 2 Gy of challenging. Consequently, the number of micronuclei (MN) in binuclear lymphocyte cells was counted as an endpoint. The results showed that the mean frequency of micronuclei in binuclear lymphocytes which have received both conditioning and challenge doses are significantly reduced in comparison to those only exposed to 2 Gy (20.46±2.13, 30.2±3.29) (P< 0.01). The results showed the existence of an *in vitro* adaptive response in lymphocyte cell exposed to low dose of gamma radiations.

Among the different physical or chemical genotoxic agents, the solar ultraviolet light and ionizing radiation are considered as natural agents affecting organisms. Some other agents are released in the environment as a result of human activity known as anthropogenic environmental pollutants. There are quite few defense mechanisms in living systems which can decrease genotoxic damage. One of them is the radio-resistance or adaptive response (AR) (1). The AR will occur when cells are exposed to very low doses of DNA-damaging agents at low dose which increases radio-resistance and makes cells less sensitive to secondary higher doses of radiation or chemicals and consequently, genotoxic damages will decrease (2). The AR has been investigated in many different organisms such as: bacteria, yeast, higher plants, insect cells, mammalian cells, human cells *in vitro*, and *in vivo* in animal models during a protracted (low dose-rate) exposure prior to an acute dose treatment (1). Adaptive behavior was found to be a characteristic feature of both mammalian and plant cells in their response to various mutagenic agents (3, 4). Adaptation to low level of alkylation, oxygen species and incorporated 3H-thymidine (3HdThd) or X-rays has been reported utilizing different biological end points (3, 5, 6). The first document published by Samson et al*.*(1977) (7) demonstrated that bacteria exhibit an AR to alkylating agents by stimulating DNA repair system. Many attempts have been made to demonstrate AR in eukaryotes(8, 9) particularly in mammalian cells (10, 11) and eventually many studies, have shown that adaptive response to radiation could occur in human lymphocytes (10, 12). Several mechanisms have been proposed for AR such as: interfering with apoptosis, enhanced DNA repair, cell repopulating events, scavenging of free radicals by adapting proteins, unknown signal transduction pathways, immunological responses etc. (13). AR is assessed using cytogenetic biomarkers like chromosome aberration (CA), micronuclei (MN), and sister chromatid exchange (SCE) (13).

In the present study, a comparative study on adaptive response using condition dose 5 cGy in human lymphocytes was performed and the effect of gamma radiation at a dose of 2 Gy on adaptive response was investigated.

## Material and Methods

*Experimental design*

In this experiment, 30 healthy, non-smoker female volunteers aged 19-35 were selected randomly among (O-RH+) blood group subjects due to the higher frequency of this blood group in Babol.

The protocol was approved by the Ethics and Scientific Research Committee of Babol University of Medical Sciences. The volunteers signed a written informed consent letter before enrolling in this study. Gamma radiation at a dose of 5 cGy was selected as condition dose (-). To deliver challenging dose, gamma radiation at a dose of 2 Gy was selected (-). Blood samples (2 ml heparinated venous blood) from each volunteer were aliquoted into 4tubes. One part was considered as control group (CTL group). A second part was considered as condition dose and received 5cGy gamma radiation (COD group). The third part was considered as challenging dose and received 2Gy gamma radiation (CD group) and the fourth part was exposed to 5cGy condition dose plus 2Gy challenge dose (COD+CD group).

*Peripheral blood lymphocytes culture*

Each blood sample aliquot was added to 4.5 ml of complete medium (RPMI-1640 supplemented by10% fetal calf serum, 2 mM L-glutamine, 100 U/ml penicillin, and 100 µg/ml streptomycin). Phytohemagglutinin (PHA) was added as mitogen to stimulate G0 lymphocytes. As suggested by Fenech et al., PHA addition time was considered as cell culture zero time point (20) and the samples were incubated in a CO2 incubator at 37 °C for 24 hours.

## Results

Mean micronuclei frequencies in lymphocytes of all four groups are summarized in Table 1.

Mean micronuclei frequency in lymphocytes in group 3 (CD group) was 30.2± 3.29, whereas, this frequency significantly decreased in group 4(p<0.001). In fact when the cells initially received 5cGy followed by 2Gy of challenging dose (group 4), the mean of micronuclei was 20.46± 2.13

## Discussion

Very low doses of DNA-damaging agents can cause adaptation of the cells against higher doses of the same agents, so the cells are less susceptible to damage by subsequent higher doses of these agents. This phenomenon is called adaptive response (AR). This issue was reported for the first time in prokaryotes by Samson and Cairn in 1977 about alkylating agents (7). Later on, many researches were carried out on mammalian cells(3, 21) with other agents such as those causing oxidative stress (e.g.; ionizing radiation)(10, 22). Radio-Adaptive-Response indicates that very low doses of ionizing radiation (few cGy) decrease genotoxic effect of a subsequent high challenge dose (23) It has been shown that optimal dose ranges for the adaptive response to ionizing radiation are 0.5-20 cGy for X-rays (12) and 1-10 cGy for 3H-TdR (6) and 1-20 cGy for Co-60 (24). In the present study ,we used 5cGy condition dose of gamma ray, according to studies carried out by A. Wojcik and H. Tuschl (15), Y. Bai et al. (24), S. M. J. Mortazavi et al. (25), S.M.J. Mortazavi et al. (26) and other studies. When the cells were initially irradiated with low doses of radiation (5cGy) followed by 2 Gy, we expected to have a higher mean of micronuclei compared to 2Gy dose alone. The results showed that the mean of micronuclei frequency significantly decreased using 5 cGy of condition dose. In the present study, we found that exposure to 5 cGy of ionizing radiation as a condition dose before higher doses of radiation can induce adaptive response.

**Table 1:** Mean frequency of micronucleie for 4 group and their standard division (1:Sham, 2:Only 0.05 Gy, 3:Only 2Gy, 4:0.05 +2 Gy)

Group	Treatment		Means of micronucleies	Std. Error	
	0.05Gy	2Gy			
1	-	-	9.06		1.66
2	24h	-	9.46		1.58
3	-	48h	30.2		3.29
4	24h	48h	20.46		2.13
